# Does Massive Bowel Resection in Newborns Affect Further Immunity in Children?

**DOI:** 10.3390/children11010114

**Published:** 2024-01-17

**Authors:** Katarzyna Sznurkowska, Anna Borkowska, Agnieszka Zagierska, Magdalena Malanowska, Maciej Zieliński, Maciej Zagierski, Piotr Trzonkowski, Marcin Łosin, Agnieszka Szlagatys-Sidorkiewicz

**Affiliations:** 1 Department of Paediatrics, Pediatric Gastroenterology, Allergology and Nutrition, Medical University of Gdańsk, Nowe Ogrody 1-6, 80-803 Gdańsk, Poland; andzia@gumed.edu.pl (A.B.); agnieszka.jankowska@gumed.edu.pl (A.Z.); maciej.zagierski@gumed.edu.pl (M.Z.); agnieszka.szlagatys-sidorkiewicz@gumed.edu.pl (A.S.-S.); 2 Department of Paediatrics, Hematology and Oncology, Medical University of Gdańsk, Dębinki 7, 80-211 Gdańsk, Poland; magdalena.malanowska@gumed.edu.pl; 3 Department of Medical Immunology, Medical University of Gdańsk, Dębinki 7, 80-211 Gdańsk, Poland; mzielinski@gumed.edu.pl (M.Z.); ptrzon@gumed.edu.pl (P.T.); 4 Department of Surgery and Urology for Children and Adolescents, Medical University of Gdansk, Dębinki 7, 80-211 Gdańsk, Poland; marcin.losin@gumed.edu.pl

**Keywords:** short bowel syndrome, lymphocyte subsets, immunoglobulins, children

## Abstract

Background: The massive resection of the small intestine leading to short bowel syndrome (SBS) deprives an organism of many immunocompetent cells concentrated in gut-associated lymphoid tissue, the largest immune organ in humans. We have aimed to access the influence of bowel resection on adaptive immunity in children, based on peripheral lymphocyte subsets and serum immunoglobulins. Methods: 15 children who underwent bowel resection in the first months of their life and required further home parenteral nutrition were enrolled into the study. Based on flow cytometry, the following subsets of lymphocytes were evaluated: T, B, NK, CD4+, C8+, and activated T cells. Results: Statistically significant differences were found for the rates of lymphocytes B, T, CD8+, and NK cells. The absolute count of NK cells was lower in the SBS group than in the control group. Absolute counts of lymphocytes, lymphocytes B, T, CD4+, and percentages of lymphocytes CD4+, and activated T cells inversely correlated with age in SBS group. Conclusions: Children with SBS do not present with clinical signs of immunodeficiency as well as deficits in peripheral lymphocyte subsets and serum immunoglobulins. The tendency of the lymphocyte subpopulations to decrease over time points out the necessity for longer follow- up.

## 1. Introduction

Short bowel syndrome (SBS) is defined as the malabsorptive condition most often caused by a massive resection of the small intestine [1]. SBS is the most common cause of intestinal failure, which is the state when an individual’s gastrointestinal function is inadequate to maintain nutrient, growth, and hydration status without intravenous or enteral supplementation [1]. In children, most cases of SBS originate during the newborn period and result from congenital anomalies or necrotizing enterocolitis [2]. Gut failure can now be successfully managed, due to prolonged parenteral nutrition in hospital and/or at home [3]. The loss of gut mucosa during resection does not only mean the loss of absorption area but also deprives the organism of many immunocompetent cells representing innate and adaptive response. Gut-associated lymphoid tissue (GALT), which is regarded as the largest immune organ in human body, contains a variety of immune cell types, particularly lymphocytes [4]. GALT plays a critical role in the development of the systemic immune response. As a primary site of antigen exposure, it primes naïve T- and B-lymphocytes, which develop into effector cells that migrate from the intestine to other sites of the body to protect against immune challenges, such as invading pathogens [5].

Although nascent Peyer’s patches are evident in the newborn, the epithelium and lamina propria are devoid of mononuclear cells. T lymphocytes migrating from the thymus rapidly populate the thymus-dependent areas of Peyer’s patches and the epithelium, but exposure to micro-organisms in the normal environment is necessary to develop the B cell population and their germinal follicles as shown by experimental studies [6]. Children in whom a large part of the bowel was resected during the neonatal period are obviously deprived of this large immune “training” area for their adaptive response. Therefore, some investigators turned towards the assessment of immunity in children with SBS. The publications, however, are sparse and focus on selected elements of immunity. To the best of our knowledge, none of the studies investigated lymphocyte populations in children with SBS. Regarding the complicated network of immune interactions and the crucial role of GALT for the development of mucosal and systemic immunity, a massive bowel resection could potentially result in immune dysfunction. Based on these notions, we aimed to answer the question if immune deficiency expressed by peripheral lymphocytes counts and serum immunoglobulins can be a long term consequence of a massive bowel resection during the newborn period.

## 2. Patients and Methods

15 patients (aged 4 months–10 years) with short bowel disease, being under the care of a nutrition outpatient clinic at the Department of Paediatrics, Pediatric Gastroenterology, Allergology and Nutrition of the Medical University of Gdansk, were enrolled in the study. All the patients underwent resection (one or more) during the neonatal period or during infancy and consequently required total parenteral nutrition, which was continued at home. Secondary resections were performed in the patients due to complications, e.g., perforation or ileus, which finally led to short bowel syndrome. In patients where intestinal atresia anastomosis was the first surgical procedure, further resections resulted from the above-mentioned complications.

The patients did not present with any signs of infection at evaluation. Body mass and length/height were routinely measured in each patient. The BMI was calculated and expressed as percentile basing on WHO Growth Standards. The patients with a BMI lower than the 5th percentile for their age were considered to be underweight. Peripheral venous blood samples (5 mL) were collected during routine clinical controls using planned venipuncture. All the patients’ caregivers had to answer the standardized list of questions concerning infections, based on the criteria for immunodeficiency developed by the Jeffrey Model Foundation [7]. These questions concerned recurrent upper respiratory infections, pneumonias and sinusitis, skin abscesses, persistent oral thrush, other severe infections such as sepsis, meningitis, and also frequent or prolonged antibiotic therapy. Catheter-related sepsis and failure to thrive were excluded from the questionnaire as they are associated with the underlying condition. The clinical characteristics of the studied group are summarized in Table 1.

12 healthy children (aged 20 months–10 years) constituted the control group. They were patients with no underlying conditions that were scheduled for elective surgery, e.g., for inguinal hernia.

Peripheral venous blood samples (5 mL) were collected via planned venipuncture together with other medical tests before surgery.

## 3. Ethics

The study was approved by the Independent Bioethical Committee of the Medical University of Gdansk and was conducted according to the principles of the Declaration of Helsinki. Informed consent was obtained from the legal guardians of all the participants included in the study.

## 4. Statistical Analysis

The significance of the differences between the studied and control groups was verified with Mann–Whitney U-test. The relationships between pairs of variables were analyzed on the basis of Spearman’s rank correlation coefficients. The results of all of the tests were considered significant at *p* < 0.05. All analyses were performed using Statistica 10 software (Stat Soft. Inc., Tulsa, OK, USA).

## 5. Antibodies, Flowcytometry

Peripheral blood lymphocytes subsets were tested using the “lyse no wash” method. Briefly, EDTA whole blood was stained with either a CD45FITC/CD4RD-1/CD8ECD/CD3PC5, a CD45FITC/CD56RD-1/CD16PE/CD19ECD/CD3PC5, or a CD3-FITC/HLA-DR-PE antibody cocktail (all from Beckman Coulter, Brea, CA, USA) according to the manufacturer’s procedures. Then, lysis was performed with the use of an Immunoprep Reagent System and a TQ-Prep Workstation (Beckman Coulter, USA). Finally, Flow-Count Fluorospheres (Beckman Coulter, USA) were added for absolute lymphocyte counting and the sample readout was performed with a Navios flow cytometer (Beckman Coulter, USA). For each sample, a minimum of 100,000 events were recorded, and CD45 gating was applied for the T/B/NK lymphocyte enumeration, while activated T cells were identified as CD3/HLA-DR double positive cells. 

## 6. Immunoglobulins

Serum immunoglobulins were determined in all of the participants with the use of the immunoturbidimetric method.

## 7. Results

The median age was 52 months for the SBS group (in a range of 4–127 months) and 51 months for the control group (in a range of 20–120 months). 

The median BMI was 15 (in a range of the 4th–28th percentiles). Only two patients presenting with a BMI lower than the 5th percentile for age were considered underweight.

The proportions and absolute counts of the lymphocyte subsets in the studied and control groups are shown in Table 2. 

**Figure 1:**
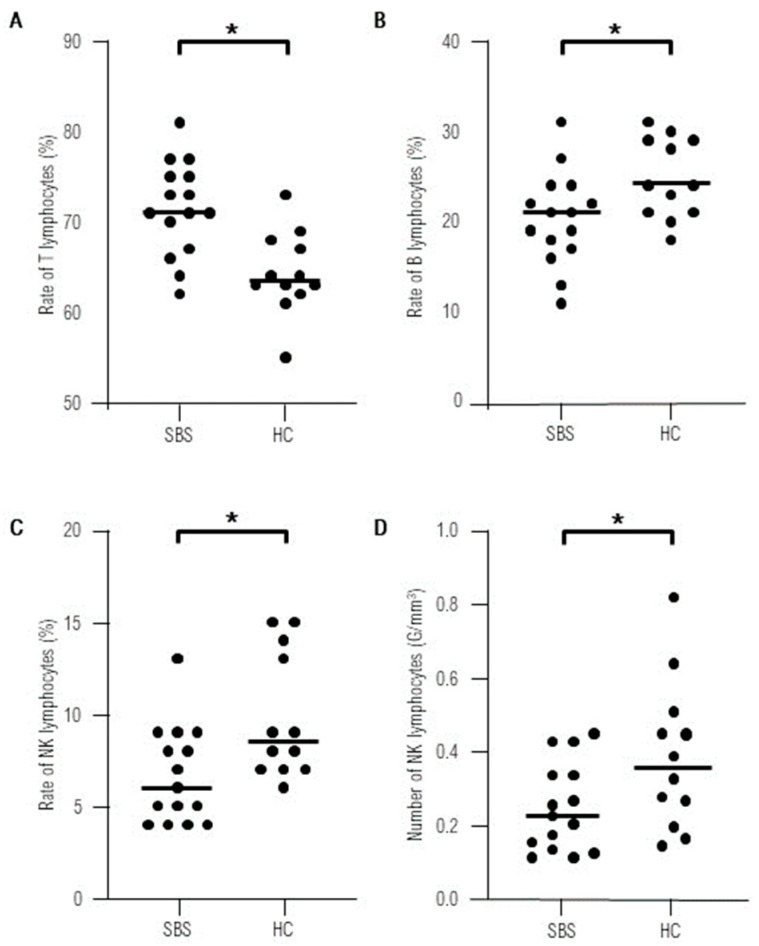
Statistically significant differences found in lymphocyte populations between the studied group of short bowel syndrome patients (SBS) and healthy controls (HC).* statistically significant-*p*-value < 0.05. (**A**) Rate of T lymphocytes in the studied and the control group. (**B**) Rate of B lymphocytes in the studied and the control group. (**C**) Rate of NK lymphocytes in the studied and the control group. (**D**) Number of T lymphocytes in the studied and the control group.

No statistically significant differences were found in the absolute numbers of lymphocytes T, B, CD4+, CD8+, and activated T cells in the study group comparing to the control group. The percentage of B lymphocytes was reduced, while the rates of T lymphocytes were higher in SBS children. We documented significantly lower absolute count and proportion of the NK cells in the SBS group compared to healthy controls (Figure 1).

No correlations between the investigated parameters and the length of the remnant bowel were noted (Table 3).

No correlations between the investigated parameters and the BMI percentile were noted (Table 3).

**Figure 2:**
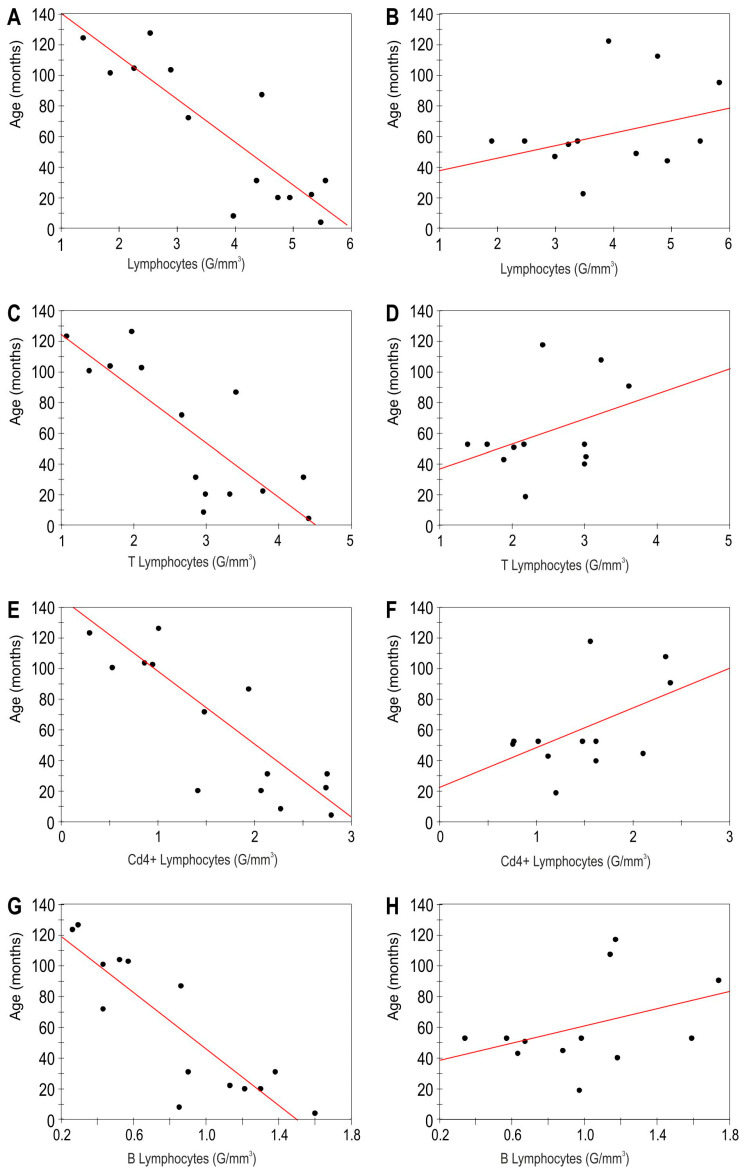
Correlation between lymphocyte subsets and age in the study group of short bowel syndrome patients, left column (**A**,**C**,**E**,**G**). Spearman’s rank correlation coefficients (R) and *p* values (*p*): (**A**) R = −0.79, *p* < 0.001 (**C**) R = −0.7, *p* < 0.001 (**E**) R = −0.81, *p* < 0.001 (**G**) R = −0.84, *p* < 0.001. Correlation between lymphocyte subsets and age in healthy controls, right column (**B**,**D**,**F**,**H**) No statistically significant results were found. Spearman’s rank correlation coefficients (R) and *p* values (*p*): (**B**) R = 0.33, *p* = 0.24 (**D**) R = 0.36, *p* = 0.21 (**F**) R = 0.42, *p* = 0.13 (**G**) R = 0.23 *p* = 0.24.

The absolute counts of lymphocytes, lymphocytes B, T, and CD4+ inversely correlated with age in the SBS group (Figure 2 and Table 3). No correlations were found with age in the healthy controls (Figure 2).

In the SBS group, the percentages of lymphocytes CD4+ inversely correlated with age (R = −0.55, *p* = 0.01) while positive correlation was found for the percentage of CD8+ cells and activated T lymphocytes (R = 0.72, *p* < 0.001 and R = 0.76, *p* < 0.001, respectively).

No statistically significant differences were found between the levels of IgA, IgM, and IgG in the studied and control groups (*p* = 0.62, *p* = 0.60, *p*= 0.24 respectively).

No patient’s caregiver reported any clinical sign of immunodeficiency in the questionnaire.

## 8. Discussion

Studies concerning immunity in children with short bowel syndrome are sparse and refer only to selected immunological aspects. Realizing the crucial role of GALT in the development of mucosal and systemic immunity, a massive bowel resection could potentially result in serious immune dysfunction. Factors that may contribute to immune disturbances in SBS include: reduction of bowel surface for GALT development, lack of normal food antigen processing, bacterial overgrowth, increased permeability, bacterial translocation, and influence of TPN on systemic immunity [8].

Some publications dealing with immunological consequences of short bowel syndrome in children have been revised by Duran, but none of them investigated the mechanism of systemic adaptive immune response [8]. Moreover, all of the analyzed publications concerned patients receiving parenteral nutrition and none of the studies related to the late immune consequences of bowel resection. The only existing study describing lymphocyte subsets in short bowel syndrome refers to adults. It documents that 10 SBS patients presented with normal values of CD3+, CD4+, and CD8+ lymphocytes, but reduced B lymphocytes in comparison with healthy individuals [9]. One has to note, however, that bowel resection in adulthood is a completely distinct state, as the removal of intestine takes place in individuals in whom immunity had previously undergone physiological development. In our cohort, no differences in the absolute counts of lymphocytes T, including CD4+ and CD8+ cells, and also B lymphocytes, were found. Only the proportions of lymphocytes T and B in the studied group significantly differed from the control group, showing a reduction in B lymphocytes’ frequency and increased rates of T lymphocytes. It is an interesting finding but we should emphasize that absolute counts are far more relevant in the interpretation of hematological data. These results do not indicate which of the mentioned findings is the primary one. It has to be noted that if the percentage of B lymphocytes decreases, the rate of T lymphocytes consequently increases and vice versa.

We have also noted the normal rate and number of activated T lymphocytes in SBS patients. This group of lymphocytes was particularly interesting as T cell activation, being the crucial process of development of the adaptive immune response, takes place mainly in mucosal-associated lymphoid tissue, including GALT. We have documented a significantly lower absolute count and proportion of NK cells in the SBS group compared to healthy controls. This population of cells was not investigated by the authors of the quoted publication. The decrease in NK cells has been observed in some congenital and acquired conditions, mostly autoimmune diseases, but has never been reported in SBS [10,11]. We have to note that, although significantly lower, NK numbers were within the normal range in all our patients and thus cannot be considered as a sign of immunodeficiency. This preliminary observation needs to be confirmed in a larger cohort. Moreover, all the subjects displayed normal levels of immunoglobulins, which is an additional argument supporting our observation that immunodeficiency is not a late effect of bowel resection. Furthermore, none of our patients demonstrated clinical signs of impaired immunity according to the criteria developed by the Jeffrey Model Foundation, which is widely accepted by clinical immunologists [7]. Although four patients had undergone catheter-related sepsis during TPN, no other severe infections were observed in the studied group. Likewise the quoted publication, our study was a retrospective assessment of peripheral lymphocytes at a different time after surgery, meaning that the studied group was not homogenous. The shortest time after resection in our cohort was four months, while the longest amounted to 10 years. It is worth noting that even the youngest subject in the studied group presented with the normal values of the studied subpopulations, which may indicate that, although reduced, the remaining bowel surface is sufficient for the lymphocytes T and B to populate the bowel and become activated. It has not, however, been clarified how long the reconstruction of GALT lasts. Some transient immunological imbalance probably appears after bowel resection as this has been demonstrated in experimental studies. Barrena et al. reported systemic immune alterations, such as a reduction in CD4+ lymphocytes and B lymphocytes, in SBS animal model seven days after surgery [12]. It may however reflect surgical stress rather than immunological imbalance caused by bowel removal, especially since analogical disturbances were also observed after other surgical interventions [13]. Another study also demonstrated changes in circulating lymphocytes numbers three weeks after surgery [14]. The short duration of these studies does not allow to conclude whether the presented abnormalities persist or rather they tend to evolve into a full immune reconstruction in experimental animals. Therefore, future prospective studies are needed to clarify the evolution of immune alterations following bowel resection in animals and in humans, especially children in whom intestinal loss takes place during the newborn or infant period.

We also examined the relationships between lymphocyte subsets and the length of the remnant bowel, but this analysis demonstrated no statistically significant results. We would like to emphasize the great heterogeneity of the group refers not only to age of the studied patients, but also to various surgical treatments. Some patients with gastroschisis and, in our cohort, also intestinal atresia had undergone many resections due to complications such as ileus or perforation, and these multiple surgeries eventually lead to short bowel syndrome. Moreover, different parts of the bowel were resected and remained in individual SBS patients. A reliable analysis would require investigating these relations in the homogenous subgroups, which is beyond the capability of the presented cohort. It is worth noting that short bowel syndrome is a rare condition, and multicenter studies could produce conclusive results.

No correlation was neither found between the studied parameters and the nutrition status of the patients expressed as the BMI percentile for age. We have to note that only two patients demonstrated BMI slightly lower than that recommended. The analysis of the relationship between the studied subsets and age revealed interesting findings. We found negative correlations for the absolute numbers of lymphocytes, lymphocytes B, T, CD4+, and the percentages of lymphocytes CD4+. We would rather expect an increase of these amounts over time as a sign of immune reconstruction and maturation. It should be noted that our subjects were operated in the first days/months of their life, so the time since resection approximately corresponds to the child’s age. We also evaluated the correlation between the lymphocyte subpopulations and the age of the children in the control group, but we noted no statistically significant results. Thus, it can be speculated that, if this trend is sustained, the patients might develop immune deficits in future, which points out to the need for a longer follow-up in SBS patients.

In conclusion, despite some weaknesses, in agreement with the main objective of the study, our results indicate that children with short bowel syndrome present neither with clinical signs of immunodeficiency nor with deficits in peripheral lymphocyte subsets and serum immunoglobulins. The decreased number of NK cells in SBS patients compared to healthy controls needs to be verified in a larger cohort. The tendency of the lymphocyte numbers to decrease over time after a resection indicates the necessity for a longer follow- up, since it can be speculated that SBS patients might develop immune deficits in future. Further research concerning other parameters of adoptive immunity in children with short bowel syndrome is warranted.

## Figures and Tables

**Table 1:** Clinical characteristics of the studied group.

Age at Evaluation (Months)	Cause of Bowel Resection	Anatomic Part and Length of Remnant Bowel	Duration of Total Parenteral Nutrition	Serum Immunoglobulins IgM; IgA; IgG (g/L)	BMI- Percentile
101	Gastroschisis	Jejunum-10 cm, colon-40 cm	Still ongoing	1.06; 1.51; 10.3	4
87	Intestinal atresia	Jejunum 30 cm, whole colon	6 years	0.81; 0.55; 8.6	12
104	Hirschsprung disease	Jejunum, ileum-80 cm, colon-0 cm	Still ongoing	0.72; 0.56; 9.22	18
72	Gastroschisis	Jejunum-50 cm, colon-45 cm	5 years	0.4; 0.41; 5.78	24
103	Intestinal atresia	Jejunum-25 cm, colon-45 cm	Still ongoing	0.54; 0.75; 12.5	18
8	Necrotising enterocolitis	Jejunum, ileum-90 cm, whole colon	Still ongoing	0.22; 0.18; 4.91	23
31	Intestinal atresia	Jejunum-45 cm, whole colon	2 years	0.4; 0.4; 5.67	28
20	Intestinal atresia	Jejunum-30 cm, whole colon	Still ongoing	0.33; 0.18; 5.03	4
22	Necrotising enterocolitis	Jejunum, ileum-80 cm, whole colon	Still ongoing	0.88; 0.18; 5.6	13
4	Intestinal atresia	Jejunum, ileum-80 cm, colon,-45 cm	Still ongoing	0.65; 0.36; 7.1	15
20	Necrotising enterocolitis	Jejunum, ileum-60 cm, colon-35 cm	Still ongoing	0.8; 0.72; 5.1	25
127	Gastroschisis	Ileum-30 cm, whole colon	5 years	0.33; 1.02; 13.75	24
124	Intestinal atresia	Jejunum-40, colon-40 cm	7 years	0.51; 0.88; 10.75	14
31	Intestinal atresia	Jejunum, ileum-60 cm, whole colon	Still ongoing	0.9; 0.72; 5.66	5
52	Intestinal atresia	Jejunum, ileum-50 cm, whole colon	3 years	0.44; 0.35; 7.64	7

**Table 2:** The proportions and absolute counts of the lymphocyte subsets expressed as the median of the interquartile range in the studied and the control groups *p*-values lower than 0.05 have been bolded.

Subpopulation/Unit	Patients with SBS (N = 15) Median(IQR)	Controls (N = 12) Median (IQR)	Statistical Difference- *p*-Value
Lymphocytes G/mm^3^	3.90 (2.5–5.0)	3.96 (2.70–4.18)	0.83
T lymphocytes %	71.0 (67.20–75.0)	63.55 (61.55–67.32)	**0.03**
T lymphocytes G/mm^3^	2.80 (1.98–3.41)	2.26 (1.92–3.04)	0.53
B lymphocytes %	21.10 (16.7–22.9)	24.95(21.95–29.10)	**0.01**
B lymphocytes G/mm^3^	0.90 (0.61–1.10)	0.98 (0.65–1.85}	0.42
CD4^+^ lymphocytes %	43.6 (34.8–48.8)	40.5 (33.5–43.8)	0.24
CD4^+^ lymphocytes G/mm^3^	1.48(1.00–2.32)	1.53 (1.08–1.85)	0.81
CD8^+^ lymphocytes %	22.7 (18.92–26.71)	19.8 (15.57–22.57	**0.04**
CD8^+^ lymphocytes G/mm^3^	0.69 (0.59–1.10)	0.77 (0.45–1.04)	0.40
NK lymphocytes %	6.1 (4.2–8.9)	8.0 (6.55–13.5)	**0.01**
NK lymphocytes G/mm^3^	0.22 (0.14–0.33)	0.34 (0.22–0.47)	**0.02**
CD4+/CD8+	1.7 (1.3–2.7)	1.77 (1.66–3.07)	0.68
CD3^+^ activated lymphocytes %	3.10 (1.7–4.1)	2.5 (1.45–4.0)	0.49
CD3^+^ activated lymphocytes G/mm^3^	0.12 (0.07–0.016)	0.13 (0.08–0.17)	0.95

Bold: *p*-Values lower than 0.05 have been bolded.

**Table 3:** Correlations between lymphocyte populations and the length of the remnant bowel, age, and BMI percentile in the study group of SBS patients. R-Spearman coefficient, *p*-value. Statistically significant results, *p*-value < 0.05, have been bolded.

Investigated Lymphocyte Subset	Correlations between Lymphocyte Subset and the Length of Remnant Bowel	Correlations between Lymphocyte Subset and Age	Correlations between Lymphocyte Subset and BMI Percentile
Lymphocytes G/mm^3^	R = −0.22 *p* = 0.37	**R = −0.79** ***p* < 0.001**	R = −0.33 *p* = 0.41
T lymphocytes %	R = −0.35 *p* = 0.15	R = 0.34 *p* = 0.16	R = 0.07 *p* = 0.79
T lymphocytes G/mm^3^	R = −0.30 *p* = 0.21	**R = −0.7** ***p* < 0.001**	R = 0.25 *p* = 0.19
B lymphocytes %	R = 0.41 *p* = 0.08	R = −0.42 *p* = 0.08	R = −0.03 *p* = 0.89
B lymphocytes G/mm^3^	R = −0.06 *p* = 0.79	**R = −0.84** ***p* < 0.001**	R = 0.16 *p* = 0.45
CD4^+^ lymphocytes %	R = −0.48 *p* = 0.06	**R = −0.55** ***p* = 0.01**	R = −0.29 *p* = 0.11
CD4^+^ lymphocytes G/mm^3^	R = −0.42 *p* = 0.08	**R = −0.81** ***p* < 0.001**	R = −0.44 *p* = 0.38
CD8^+^ lymphocytes %	R = 0.17 *p* = 0.48	**R = 0.72** ***p* < 0.001**	R = −0.31 *p* = 0.44
CD8^+^ lymphocytes G/mm^3^	R = 0.12 *p* = 0.62	R = −0.34 *p* = 0.16	R = −0.15 *p* = 0.77
NK lymphocytes %	R = 0.22 *p* = 0.38	R = 0.35 *p* = 0.15	R = 0.05 *p* = 0.84
NK lymphocytes G/mm^3^	R = −0.10 *p* = 0.69	R = −0.46 *p* = 0.06	R = −0.14 *p* = 0.61
CD3^+^ activated lymphocytes %	R = 0.12 *p* = 0.62	**R = 0.76** ***p* < 0.001**	R = 0.24 *p* = 0.38
CD3^+^ activated lymphocytes G/mm^3^	R = −0.17 *p* = 0.48	R = 0.17 *p* = 0.47	R = 0.05 *p* = 0.85

Bold: Statistically significant result have been bolded.

## Data Availability

The data presented in this study are available on request from the corresponding author. The data are not publicly available due to personal privacy.

## Abbreviations

SBS—short bowel syndrome, GALT—gut-associated lymphoid tissue, CD—cluster of differentiation, NK—natural killers.
